# Leaf‐footed bugs possess multiple hidden contrasting color signals, but only one is associated with increased body size

**DOI:** 10.1002/ece3.6468

**Published:** 2020-07-28

**Authors:** Zachary Emberts, Christine W. Miller, Chelsea Skojec, Rachel Shepherd, Colette M. St. Mary

**Affiliations:** ^1^ Department of Biology University of Florida Gainesville Florida USA; ^2^ Entomology & Nematology Department University of Florida Gainesville Florida USA

**Keywords:** autotomy, body size, Coreidae, deimatic display, eyespots, flash display, hemipteran, hidden contrasting coloration, predation, prey

## Abstract

Antipredatory displays that incorporate hidden contrasting coloration are found in a variety of different animals. These displays are seen in organisms that have drab coloration at rest, but when disturbed reveal conspicuous coloration. Examples include the bright abdomens of mountain katydids and the colorful underwings of hawk moths. Such hidden displays can function as secondary defenses, enabling evasion of a pursuant predator. To begin to understand why some species have these displays while others do not, we conducted phylogenetic comparative analyses to investigate factors associated with the evolution of hidden contrasting coloration in leaf‐footed bugs. First, we investigated whether hidden contrasting coloration was associated with body size because these displays are considered to be more effective in larger organisms. We then investigated whether hidden contrasting coloration was associated with an alternative antipredatory defense, in this case rapid autotomy. We found that leaf‐footed bugs with hidden contrasting coloration tended to autotomize more slowly, but this result was not statistically significant. We also found that the presence of a body size association was dependent upon the form of the hidden color display. Leaf‐footed bugs that reveal red/orange coloration were the same size, on average, as species without a hidden color display. However, species that reveal white patches on a black background were significantly larger than species without a hidden color display. These results highlight the diversity of forms that hidden contrasting color signal can take, upon which selection may act differently.

## Graphical abstract

We found that larger species of leaf‐footed bugs are more likely to have flash displays that reveal white patches on a black background. However, we also found that species with red/orange displays are the same size, on average, as species without hidden color displays . These results suggest that selection differs between forms of hidden displays, which could explain why hidden contrasting coloration is associated with larger body sizes in some clades but not others.

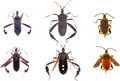

## 1 INTRODUCTION

Many of the morphological and behavioral traits observed throughout Animalia have been selected for by predation. Examples include the elongated hind wings of luna moths (Barber et al., 2015), the barbed quills of porcupines (Cho et al., 2012; Roze, 2009), and the death‐feigning behavior of red flour beetles (Miyatake et al., 2004). Theory suggests that traits such as these have evolved because they increase an individual's chance of avoiding or escaping a predator. Another potential antipredatory trait, one that has not received a great deal of attention, is hidden contrasting coloration. Hidden contrasting color patterns are found in species that are inconspicuously colored while at rest, but, when disturbed or in motion, reveal hidden coloration that may ward off predators. These signals can be divided into two main defenses, flash displays and deimatic displays. A flash display is the use of otherwise concealed contrasting color signals while in motion (Edmunds, 2008). This defense may be effective in evading predation by confusing the predator of its location, making it difficult to estimate its future trajectory (Loeffler‐Henry, Kang, Yip, Caro, & Sherratt, 2018; Murali, 2018). It is hypothesized that flash displays may also function to frighten or surprise predators, causing them to abandon or be unsuccessful in their pursuit (Murali, 2018). These displays can be seen in the colorful underwings of a variety of moths, namely sphingid and noctuid moths, as well as the hindwings of some orthopterans, mantids, and cicadas (Loeffler‐Henry, Kang, & Sherratt, 2019). Unlike flash displays, where an individual reveals color while in motion (i.e., involves rapid locomotion), deimatic displays often reveal conspicuous coloration while stationary (or with more limited locomotion; Umbers et al., 2017; Umbers, Lehtonen, & Mappes, 2015). The bright abdomens of mountain katydids are a great example (Umbers & Mappes, 2015). When at rest, these katydids are cryptically colored, but when disrupted, they open their wings, revealing a contrasting color pattern (Umbers & Mappes, 2015). This sudden behavior, accompanied by the colored abdomen, elicits a startle response in the predator, which increases the katydid's likelihood of surviving the predation event (Umbers et al., 2019).

Hidden contrasting coloration has evolved in a variety of different clades, but not all taxa within these clades possess the defensive signal. Previous research on hidden contrasting color patterns has shown that there may be an association with larger body sizes (Kang, Zahiri, & Sherratt, 2017; Loeffler‐Henry et al., 2019). Larger insects are more easily detected by avian predators (Remmel & Tammaru, 2009), potentially making a secondary defense such as hidden coloration more valuable. Additionally, color defenses may be more effective in larger prey than in smaller prey, as larger individuals may look more threatening when using the display (e.g., Hossie, Skelhorn, Breinholt, Kawahara, & Sherratt, 2015). A positive association between body size and hidden contrasting coloration is found in Orthoptera, Mantidae, Phasmatidae, and Saturniidae, but this association is not ubiquitous (Loeffler‐Henry et al., 2019). For instance, no such association is observed in the Sphingiidae clade (Loeffler‐Henry et al., 2019). Although these patterns are compelling, they also raise additional questions. For example, given that contrasting coloration is associated with body size in some cases but not others, what factors might explain the exceptions?

**FIGURE 1:**
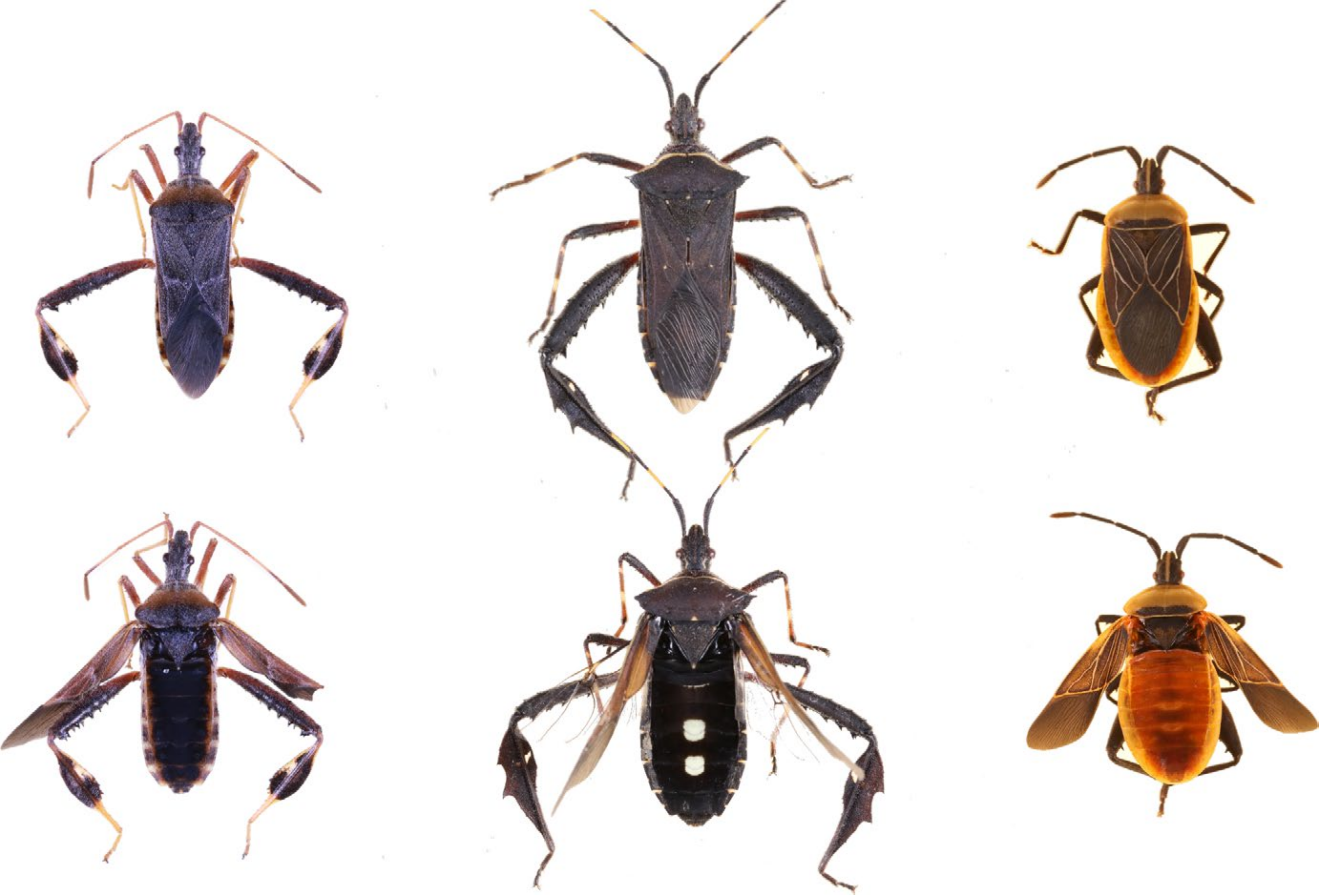
Select leaf‐footed bugs in their resting state (top row) and their flight/defense revealing state (bottom row). From left to right: *Narnia femorata*, which has a non‐contrasting abdomen, *Leptoglossus gonagra*, which has a contrasting black and white patch abdomen, and *Chelinidea vittiger*, which has a contrasting red/orange abdomen

To better understand the generality of hidden contrasting coloration being associated with larger body sizes, first we investigate this association in the leaf‐footed bug clade (Insecta: Hemiptera: Coreidae). Within this clade, there are at least two hidden color displays, a black and white patch color pattern and red/orange coloration (Figure 1). There is strong evidence to suggest that both a black and white color pattern and red/orange coloration can have antipredatory benefits (Cott, 1940; Ruxton, Sherratt, & Speed, 2004). In leaf‐footed bugs, these warning displays are found on the dorsal side of their abdomen. The location of these warning displays is important because this portion of the body is often concealed (Figure 1). When leaf‐footed bugs, and hemipterans in general, are in their resting state (i.e., non‐flying state), they fold their wings over their abdomen. As a result, the color and coloration patterns on the forewings are most frequently observed (Figure 1). However, when individuals transition their wings into their flying position a new color pattern is revealed. Since these visual signals are behaviorally dynamic, as opposed to always being displayed, they fall within the hidden contrasting color display framework (Skelhorn, Holmes, & Rowe, 2016; Umbers et al., 2015; Umbers & Mappes, 2016). Moreover, because their hidden color is only revealed while in flight, these are flash displays. However, like other clades with hidden contrasting coloration, this trait is not found in all species within this group. Thus, our first aim of this study is to determine whether hidden contrasting coloration in leaf‐footed bugs is associated with larger species and to determine whether such an association is dependent upon the form of the hidden color display.

Leaf‐footed bugs are also well known for another antipredatory defense, autotomy, which is self‐induced appendage loss (Emberts, St. Mary, Herrington, & Miller, 2018; Emberts, Miller, Kiehl, & St. Mary, 2017; Emberts, St. Mary, & Miller, 2016). Autotomy is a secondary defense (i.e., a defense to evade a pursuant predator; Edmunds, 1974) used by a variety of species to escape predation and has been observed in arthropods, cephalopods, lizards, and amphibians (Emberts, Escalante, & Bateman, 2019). Because sacrificing an appendage is often costly, it is hypothesized that the evolution of other antipredatory defenses should reduce selection for rapid autotomy. However, there is limited evidence to support this hypothesis (Bateman & Fleming, 2009). The strongest example comes from a study that compared differences in the frequency of autotomy between two sympatric gecko species and found that the more aggressive species, which actively fights back against predators, had a lower rate of autotomy (Dial, 1978). Although the ability to autotomize is ubiquitous in leaf‐footed bugs, there is extensive variation in the rate of autotomy such that only a fraction of the species can autotomize quickly enough to escape predation (Emberts et al., 2020). Thus, the second aim of this study is to investigate whether the evolution of hidden contrasting coloration, a secondary defense against predation, is associated with the speed at which species autotomize. We predicted that species with hidden contrasting coloration would be larger and would autotomize their hind limbs more slowly.

## 2 METHODS

**FIGURE 2:**
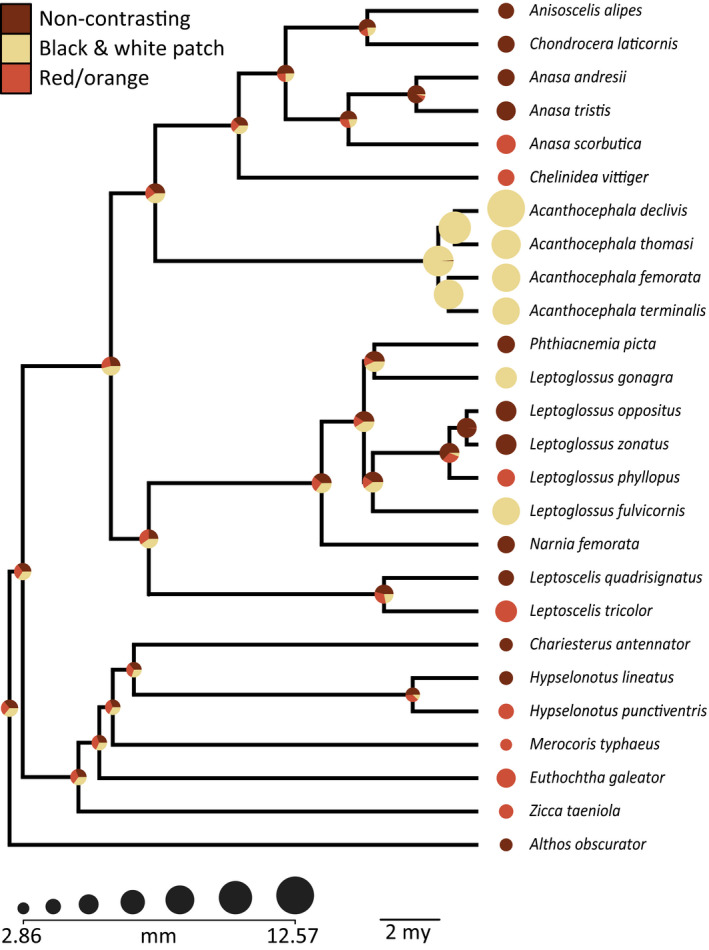
The evolution of body size and flight/defense revealing states in leaf‐footed bugs. Circle color corresponds to flight/defense revealing states, while the circle size corresponds to body size (PW, mm). Node circles visualize the estimated ancestral body size and flash/defense revealing state

**FIGURE 3:**
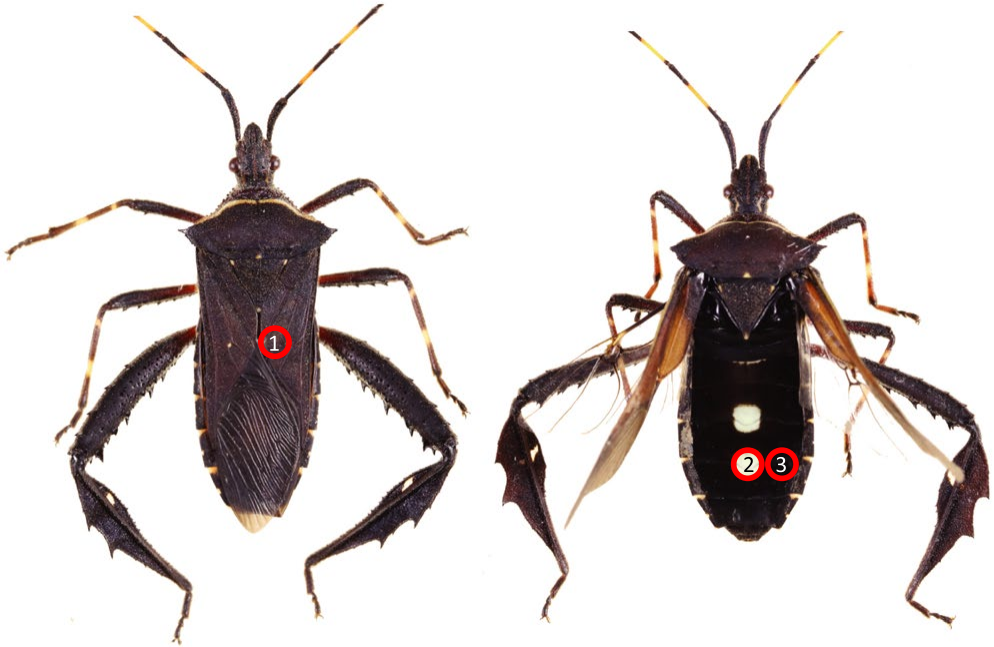
Locations where RGB coloration was quantified: the corium (1), the dorsal‐medial abdomen (2), and the dorsal‐lateral abdomen (3). Photographed images are of *Leptoglossus gonagra*. The same three locations were used to quantify coloration for all individuals

**FIGURE 4:**
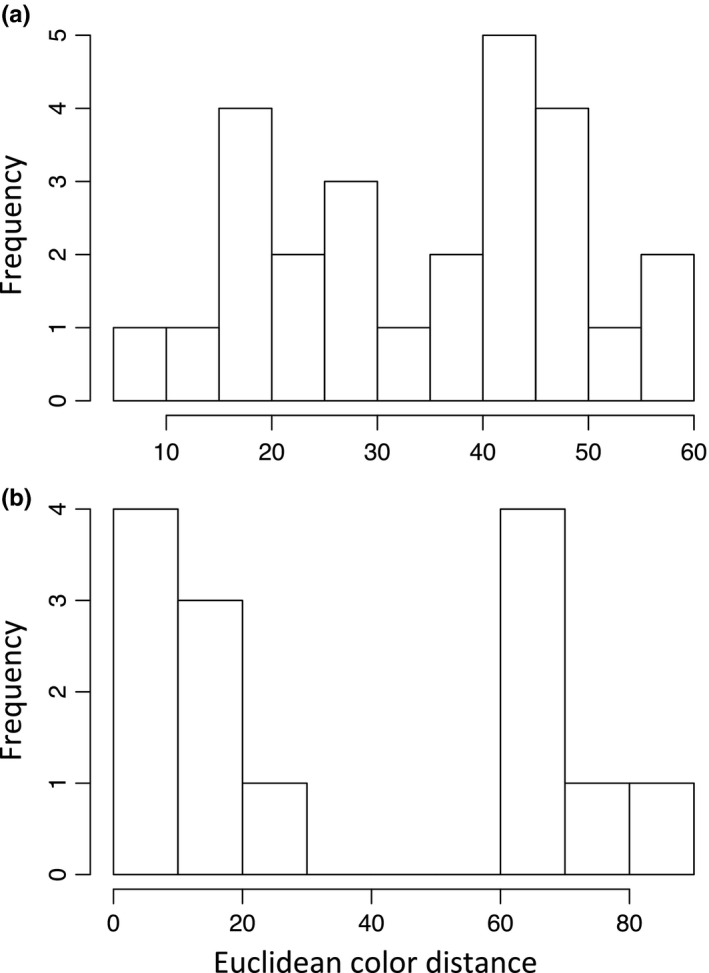
We assumed that the distribution of our Euclidean color distances would have two modes for our k‐means clustering analyses. Visualization of our data confirmed that this was a reasonable assumption. Subfigure a is the average Euclidean color distance between the corium and the dorsal‐medial abdomen (*n* = 26). Cluster means were estimated to be at a Euclidean color distance of 21.3 and 46.6. Total between cluster sum of squares was 4,116 out of a total sum of squares of 4,948. Subfigure b is the average Euclidean color distance between the dorsal‐medial abdomen and the dorsal‐lateral abdomen (*n* = 14). Cluster means were estimated to be at a Euclidean color disstance of 10.9 and 70.0. Total between cluster sum of squares was 11,985 out of a total sum of squares of 12,532

To investigate our hypotheses, we used a subclade of leaf‐footed bugs for which there is an existing phylogenetic hypothesis, body size measurements, and autotomy data (data from Emberts et al., 2020). We specifically selected this subclade because we had access to almost every species that was represented in the phylogeny (*n* = 26 species; Figure 2) and our previous knowledge suggested that this clade captures the diversity of flight/defense revealing states observed throughout the larger leaf‐footed bug clade. Thus, this clade included taxa with non‐contrasting abdomens, those with black and white patch abdomens, and those with red/orange abdomens (Figure 1). For each species, we determined whether hidden contrasting coloration was present and, if so, the form of its display (i.e., black and white patch color pattern or red/orange coloration). To do this, we photographed up to five individuals per species (median: 5, range: 1–5) with a Canon EOS 50D Camera. Each individual was frozen and then photographed with their wings in their resting position and in their flying position (Figure 1). These photographs were taken inside, under consistent artificial lighting. We then quantified RGB coloration of the corium (i.e., the color displayed at rest), the dorsal‐medial abdomen (i.e., part of the revealed color), and the dorsal‐lateral abdomen (i.e., another part of the revealed color; Figure 3) for each individual in Adobe Photoshop. RGB coloration was then converted to LAB color space. To determine whether or not a species had hidden contrasting coloration, we calculated the average Euclidean color distance between the corium (i.e., the color displayed at rest) and the dorsal‐medial abdomen (i.e., the most conspicuous color revealed) for each species (Figure 3). Then, following Loeffler‐Henry et al. (2019), we conducted a k‐means clustering analysis, assuming two modes (Figure 4), to determine whether a species did or did not have hidden contrasting coloration. Those that were considered to have hidden contrasting coloration were further investigated to objectively determine whether their revealed display should be classified as uniform or patterned. We did this by calculating the mean Euclidean color distance between the dorsal‐medial location and the dorsal‐lateral location of the abdomen for each species (Figure 3). Again, we conducted a k‐means clustering analysis, still assuming two modes (Figure 4), to determine whether a species had a similar color pattern across their abdomen. The first mode captures the red/orange abdomen coloration, while the second mode captures those with contrasting color patterns (i.e., black and white patch abdomen; Figure 4).

For the purposes of this study, we pruned the 62‐tip leaf‐footed bug phylogeny from Emberts et al. (2020), which was built from a concatenated alignment of 567 loci (Emberts et al., 2020, Figure 2). The phylogenetic reconstruction in Emberts et al. (2020) was done in a maximum likelihood framework as implemented in RAxML (Stamatakis, 2014), and the tree was dated using penalized likelihood as implemented in treePL (Smith & O’Meara, 2012) with four fossil calibrations. In the same study, the authors reported data on body size and latency to autotomize for each taxon. The mean and median latency to autotomize (i.e., two measures for the rate of autotomy) for each species was estimated by collecting autotomy data on up to 84 individuals per species. Autotomy trials were conducted by grasping an individual's hind right leg with constant pressure (i.e., reverse action) forceps, while the individual was in contact with a piece of wood for up to 1 hr (Emberts et al., 2020). Those that did not autotomize within an hour were assigned as taking 3,600 s to autotomize (as opposed to removing them) because evidence suggests that these individuals would have likely autotomized, given more time (see Emberts et al., 2020). Pronotal width, a body size proxy for this clade (Emberts et al., 2020; Procter, Moore, & Miller, 2012), was measured to the nearest micrometer for each of these individuals as well.

To determine whether hidden contrasting coloration (presence/absence, independent variable) was associated with body size (continuous, dependent variable), first we had to identify the best model of trait evolution. We specifically investigated whether Brownian motion (BM), Ornstein–Uhlenbeck (OU), or early burst (EB) was the best model of trait evolution for our body size data using geiger v 1.2.2 (Harmon, Weir, Brock, Glor, & Challenger, 2008). Akaike information criterion identified the best model as a BM model of trait evolution (Table 1). Thus, we conducted a phylogenetic generalized least square (PGLS) analysis assuming a BM model of trait evolution as implemented in phytools v 0.6–60 (Revell, 2012). Then, we conducted post hoc analyses where we ran an individual PGLS for all pairwise combinations of flight/defense revealing states (i.e., three additional PGLS models) to identify whether a specific flight/defense revealing state was responsible for driving any association. Thus, for one PGLS model, we removed species with contrasting black and white patch abdomens from the dataset and investigated whether there was any body size differences between species with non‐contrasting abdomen compared to those with contrasting red/orange abdomens. Our second PGLS model removed species with contrasting red/orange abdomens from the complete dataset, while our third PGLS model removed species with non‐contrasting abdomens. Body size was log_e_‐transformed for these analyses to improve linearity, normality, and homoscedasticity.

**TABLE 1:** Akaike information criterion (AIC) revealed an OU model as the best model for the autotomy data and a BM model as the best model for the body size data

Model	Body size AIC	Mean autotomy AIC	Median autotomy AIC
BM	8.9	124.4	131.6
OU	9.1	118.7	125.7
EB	10.9	126.4	133.6

Abbreviations: BM, Brownian motion; EB, early burst; OU, Ornstein–Uhlenbeck.

To determine whether hidden contrasting coloration was associated with autotomy, we first identified whether autotomy evolved via BM, OU, or EB and found that it evolves via an OU model. Next, we size corrected our latency to autotomize variables (i.e., mean and median) because body size can explain variation in the latency to autotomize (Emberts et al., 2020). We did this by conducting a PGLS analysis assuming an OU model with autotomy (continuous, log_e_‐transformed) as the dependent variable and body size (untransformed pronotal width) as the independent variable and extracted the model residuals. We then investigated whether these residuals (dependent variable) were associated with hidden contrasting coloration (binary, independent variable).

We also calculated Cohen's *d*, a standardized effect size estimation, for each of our analyses (Cohen, 1988). Cohen's *d* was calculated by taking the difference between two means and dividing it by the pooled standard deviation of the two groups. Effect size estimation between 0.20 and 0.49 is considered to be small, that between 0.50 and 0.79 is considered to be medium, and that greater than 0.80 is considered to be large (Cohen, 1988). All analyses were conducted in R v 3.6.0 (R Core Team, 2019).

## 3 RESULTS

**FIGURE 5:**
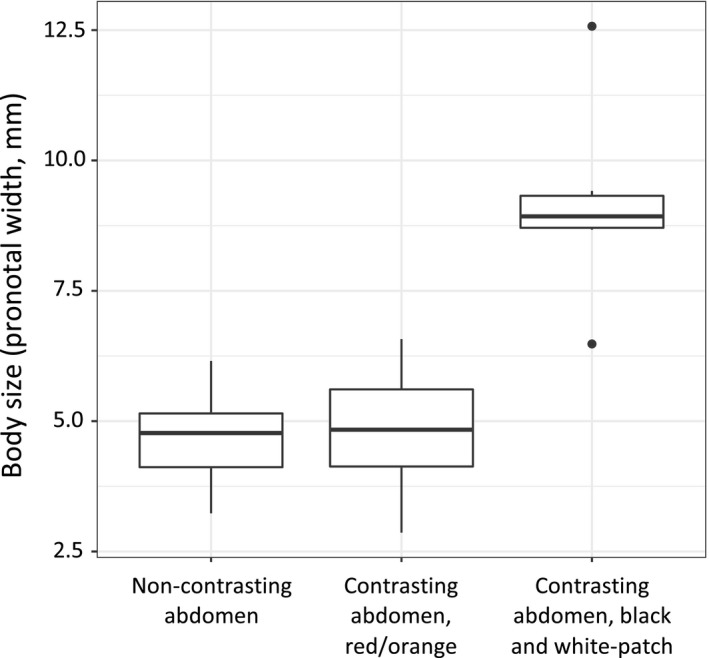
Leaf‐footed bug species that reveal white patches on a black background are larger than species that reveal red/orange coloration and those without hidden color displays. Furthermore, species that reveal red/orange coloration are similar in size to species without hidden color displays

Hidden contrasting coloration was found throughout the leaf‐footed bug clade. Of the 26 species we investigated, 12 had non‐contrasting abdomens and 14 had contrasting abdomens. Of those that had hidden contrasting coloration, six revealed white patches on a black background and eight revealed red/orange coloration. Larger leaf‐footed bugs appeared to be more likely to have one of these hidden color displays (*t*_24_ = 1.076, *p* = .293; estimated coefficient ± *SE* = 0.092 ± 0.086; Cohen's *d* effect size estimation = 0.91). This pattern, however, was almost completely driven by hidden contrasting coloration in the form of white patches on a black background; species that possess this signal are larger than those with non‐contrasting coloration (*t*_16_ = 2.982, *p* = .009; estimated coefficient = 0.432 ± 0.145, Cohen's *d* = 3.17) and those with contrasting red/orange coloration (*t*_12_ = 2.411, *p* = .033; estimated coefficient = 0.475 ± 0.197; Cohen's *d* = 2.76; Figure 5). Moreover, those with contrasting red/orange abdomen were similar in size to those with non‐contrasting abdomens (*t*_18_ = 0.187, *p* = .854, estimated coefficient = 0.012 ± 0.066, Cohen's *d* = 0.06; Figure 5). Species that possessed hidden contrasting coloration also took twice as long, on average, to autotomize their hind legs (1,690s compared to 802s). However, after including body size and phylogenetic relationship into the analysis, this difference was not supported statistically (mean latency to autotomize: *t*_24_ = 1.870, *p* = .074; estimated coefficient = 1.506 ± 0.805, Cohen's *d* = 0.75; median latency to autotomize: *t*_24_ = 1.354, *p* = .189, estimated coefficient = 1.252 ± 0.925, Cohen's *d* = 0.71).

## 4 DISCUSSION

We found that larger species of leaf‐footed bugs are more likely to have flash displays that reveal white patches on a black background (Figure 5). However, we also found that species with contrasting red/orange abdomens are the same size, on average, as species with non‐contrasting abdomens (Figure 5). These results suggest that selection differs between forms of hidden displays, which could explain why hidden contrasting coloration is associated with larger body sizes in some clades but not others.

There are several reasons why larger leaf‐footed bugs may be more likely to possess flash displays that are patterned (white patches on black background) rather than uniformed (red or orange). One possibility is that predators perceive the white patches in this clade as eyespots (Stevens, Hardman, & Stubbins, 2008, but see De Bona, Valkonen, López‐Sepulcre, & Map pes, 2015). Research has demonstrated that eyespots can effectively interrupt and, in some cases, prevent a predation event (De Bona et al., 2015; Hossie & Sherratt, 2013; Stevens, 2005). Moreover, previous experiments on eyespots have shown that these spots provide a protective advantage for larger caterpillars (4 cm body length), but not smaller ones (2 cm body length; Hossie et al., 2015). Such differential selection is considered to be the main mechanism promoting a positive association between eyespots and body size in hawkmoth caterpillars (Hossie et al., 2015). This same mechanism could also be driving this association in leaf‐footed bugs as those with white patches were approximately 2–4 cm long, whereas those without white patches were smaller. Along these lines, predation in leaf‐footed bugs may be associated with body size, and certain flash display may be more effective against certain predators. For example, larger species of insects may face more predation from avian than invertebrate predators (e.g., Remmel, Davison, & Tammaru, 2011), and the white patches in this clade may be more effective against birds. Red/orange coloration, on the other hand, can effectively deter both avian and invertebrate predators (Bowdish & Bultman, 1993; Dell'Aglio, Stevens, & Jiggins, 2016; Taylor, Maier, Byrne, Amin, & Morehouse, 2014). This could explain why red/orange coloration is found throughout a wide range of leaf‐footed bug sizes. Regardless of the mechanism that is responsible for driving a body size association for some displays, but not others, this study highlights that hidden contrasting color signals can take on a diversity of forms and that selection may differentially act upon them.

Our finding that leaf‐footed bugs with contrasting red/orange abdomens are the same size, on average, as those with non‐contrasting abdomens are inconsistent with previous research. For example, in simulated studies that use human predators, flash displays that reveal red coloration increase survivorship in larger individuals, but not smaller ones (Bae, Kim, Sherratt, Caro, & Kang, 2019). Such differential selection should result in larger species being more likely to have flash displays that reveal red/orange coloration, which we did not find (Figure 5). This discrepancy could simply be explained by differences in the natural predators of a species and the limits of the predators’ vision, such as color perception and visual acuity (Caves, Brandley, & Johnsen, 2018; Kemp et al., 2015). It could also be argued that this inconsistency is driven by the conspicuousness of the flash displays, that is, how well the signal stands out within a given environment. It is possible that the red abdomens of leaf‐footed bugs are inconspicuous in their natural environment, in which case the display would not provide a survival advantage (Bae et al., 2019). Although we did not quantify conspicuousness here, we find this hypothesis to be unlikely given our personal experience collecting these bugs in the field. Another possibility is that red coloration may be costly to produce in this clade (see Hill, 1996 for the cost of red coloration in birds), and the additional signaling area may make red coloration too costly for larger species. Moreover, there could be species specific constraints inhibiting an association between size and red/orange coloration at the macroevolutionary level. For example, some leaf‐footed bug species may be unable to produce red coloration. Future studies should continue to investigate why flash displays that reveal red coloration are associated with larger taxa in some clades, but not others. Studies that specifically investigate how effective these signals are against different classes of predators as well as those that quantify the costs associated with these signals would provide valuable insight.

It is unclear from our results whether flash displays negatively correlate with fast autotomy. This uncertainty can largely be attributed to a small sample size because our effect size estimation suggests that flash displays have a moderate effect on autotomy (i.e., 0.71 and 0.75). Although it is unclear whether autotomy and flash displays have a negative correlation, our results clearly indicate that they do *not* have a positive correlation. That is to say, it is quite rare for a leaf‐footed bug to autotomize its hind limb quickly and to have a flash display. This result is consistent with (although it provides no additional support for) the hypothesis that the presence of other antipredatory defenses should reduce selection for rapid autotomy given the costs associated with sacrificing an appendage (Arnold, 1988; Bateman & Fleming, 2009).

Much remains unknown about the evolutionary influence that antipredatory traits can have upon one another. In leaf‐footed bugs, some individuals possess both flash displays and the ability to rapidly autotomize their limbs, but possessing both antipredatory defenses was rare. Moreover, increased body size was associated with some flash displays, but not others. Future works should try to understand the generality of these patterns, as well as the mechanisms behind them. Both orthopterans and phasmids possess hidden contrasting color displays (Loeffler‐Henry et al., 2019) and are known to autotomize (Fleming, Muller, & Bateman, 2007) making them ideal clades for further investigation.

## CONFLICT OF INTEREST

We have no conflict of interests to declare.

## AUTHOR CONTRIBUTIONS

**Zachary Emberts:** Conceptualization (lead); data curation (lead); formal analysis (lead); investigation (supporting); methodology (lead); visualization (lead); writing–original draft (lead); writing–review and editing (equal). **Christine W. Miller:** Conceptualization (supporting); funding acquisition (equal); writing–review and editing (equal). **Chelsea Skojec:** Investigation (supporting); writing–original draft (supporting); writing–review and editing (equal). **Rachel Shepherd:** Investigation (lead); writing–review and editing (equal). **Colette M. St. Mary:** Conceptualization (supporting); funding acquisition (equal); writing–review and editing (equal).

## Data Availability

Data can be found in Dryad Digital Repository (https://doi.org/10.5061/dryad.wdbrv15kk).
